# Soybean Seeds: A Practical Host for the Production of Functional Subunit Vaccines

**DOI:** 10.1155/2014/340804

**Published:** 2014-04-14

**Authors:** Laura C. Hudson, Renu Garg, Kenneth L. Bost, Kenneth J. Piller

**Affiliations:** ^1^Soymeds, Inc., Davidson, NC 28036, USA; ^2^CSIR-Institute of Microbial Technology, Sector 39A, Chandigarh 160036, India; ^3^Department of Biology, University of North Carolina at Charlotte, 9201 University City Boulevard, Charlotte, NC 28223, USA

## Abstract

Soybean seeds possess several inherent qualities that make them an ideal host for the production of biopharmaceuticals when compared with other plant-based and non-plant-based recombinant expression systems (e.g., low cost of production, high protein to biomass ratio, long-term stability of seed proteins under ambient conditions, etc.). To demonstrate the practicality and feasibility of this platform for the production of subunit vaccines, we chose to express and characterize a nontoxic form of *S. aureus* enterotoxin B (mSEB) as a model vaccine candidate. We show that soy-mSEB was produced at a high vaccine to biomass ratio and represented ~76 theoretical doses of human vaccine per single soybean seed. We localized the model vaccine candidate both intracellularly and extracellularly and found no difference in mSEB protein stability or accumulation relative to subcellular environment. We also show that the model vaccine was biochemically and immunologically similar to native and recombinant forms of the protein produced in a bacterial expression system. Immunization of mice with seed extracts containing mSEB mounted a significant immune response within 14 days of the first injection. Taken together, our results highlight the practicality of soybean seeds as a potential platform for the production of functional subunit vaccines.

## 1. Introduction

The use of transgenic plants to express recombinant proteins has gained popularity over the past decade and represents a growing segment in the pharmaceutical industry. Currently, the bulk of biopharmaceuticals are produced in recombinant microbe expression systems or insect and mammalian cell cultures. However, as with all protein expression systems, there are advantages and disadvantages to these systems which are described in several review articles [1–3]. Some of these limitations include the types of proteins that can be produced and in the posttranslational processing that can be achieved making production costs prohibitively high. Based on these limitations, an increased demand for biopharmaceuticals will require improved and cost effective manufacturing practices and practical transportation methods for a global community.

As an alternative to traditional systems, a number of pharmaceuticals have been successfully produced in various plant-based expression systems. Although these plant systems offer great potential, they too present several challenges. Many crop systems used to date have a low protein content which can increase the overall production costs since purification expenses are typically inversely proportional to final target protein concentration in plant biomass. Therefore, crops with higher protein content and a compact biomass are more cost effective for molecular farming. When it comes to express large amounts of a pharmaceutical protein in a plant host, soybean should be considered as a practical alternative. The soybean system has many distinct advantages when compared with existing expression systems. For example, soybeans contain ~40% protein by dry mass and therefore represent one of the richest natural sources of protein known. Given this high protein content, it is possible to express large amounts of transgenic protein in a single soybean seed. Furthermore, with typical transgenic expression levels of 1–4% of total soluble protein (TSP), there are few, if any, host systems that can produce such levels of foreign protein based on weight. Second, soybean is a relatively easy and inexpensive plant to grow making the production of biopharmaceuticals in soybeans extremely cost effective. Another advantage of soybean is the proven stability of proteins in dry mass over extended periods of time, suggesting that pharmaceuticals could be shipped as crushed seed or processed powder and stored under ambient conditions, thus eliminating any requirement for a cold chain. Soybean also possesses the necessary machinery for eukaryotic posttranslational modification [4] and is capable of generating large and complex recombinant proteins (>600 kDa) that are often recalcitrant to expression in traditional expression systems [5]. Given these advantages, soybean represents a practical host for the production of proteins for numerous applications.

Soybean-based vaccines, in particular, offer specific advantages over vaccines produced in other, more conventional systems. For example, soy-based vaccines could either be formulated into consumables for oral delivery or purified for injection or other downstream uses. In an effort to demonstrate the practicality of soybean seeds as a host system for manufacturing protein-based vaccine candidates we chose to express a nontoxic form of Staphylococcal enterotoxin B (SEB) as a model vaccine candidate. SEB is a well-characterized, superantigen-like exotoxin produced by the bacteria* Staphylococcus aureus*. SEB mediates its toxicity by linking MHC class II molecules with T cell receptors outside of the antigen binding site [6]. Clinical symptoms of SEB poisoning include anorexia, nausea, vomiting, and diarrhea. Three-dimensional structures of SEB and its complex with MHC class II molecules have been elucidated [7, 8] and several biochemical studies have offered clues to the biologically important regions of this protein [9–12]. While SEB remains a CDC Category B toxin, there is still no vaccine for SEB poisoning in the market.

Due to the inherent superantigen properties of SEB, the native toxin cannot be used as a practical vaccine antigen. However, mutated forms of the protein that remove superantigenicity while leaving immunogenic capacity intact should serve as a viable vaccine option. Such alterations can be accomplished using chemical treatment or genetic manipulation to introduce site specific mutations [13, 14]. Various mutagenesis studies have identified important mutations that reduce or eliminate biological activity of the wild-type toxin while retaining immunogenic epitopes that elicit protective antibody responses [10, 12, 15]. Specifically, single mutations of key residues in the hydrophobic binding loop (L45R), polar binding pocket (Y89A), and disulfide loop (Y94A) in recombinant forms of SEB eliminated binding to the MHC class II receptor [15] but did not disrupt native structure and generated effective immune responses. This triple mutant form of SEB (mSEB) possessed greatly diminished biological activity and was reported to be tolerated as a vaccine in both mice and nonhuman primates. The triple mutant also offered protection to immunized animals when challenged with native SEB (nSEB) [14–16]. Synthetic mSEB has also been used as a model antigen and overexpressed in tobacco (NT1) cells using a geminivirus-based replicon system [17].

In this study we engineered a model vaccine candidate to demonstrate the practicality of soybean as a platform for the production of vaccine candidates and other therapeutics. Two well-characterized plant promoters were used to target expression to seeds, and different signal peptides were included to evaluate accumulation in different subcellular locations. We found that all recombinant forms of the model mSEB vaccine were expressed at a high vaccine to biomass ratio and were accurately processed by the plant machinery. We show that a soy-mSEB vaccine candidate was biochemically equivalent to, and exhibited immunological properties that were analogous to, those exhibited by* E. coli*-derived mSEB and native SEB. Functionality of soy-mSEB was shown in groups of mice immunized with the model vaccine candidate. Taken together, these findings demonstrate the practicality of soybean as a cost-effective host for the production of important vaccine candidates.

## 2. Materials and Methods

### 2.1. Seed-Specific SEB Expression Cassette Design and Construction

Soybean codon optimized mutant SEB genes containing sequences encoding the native SEB N-terminal signal peptide sequence and the native soybean glycinin N-terminal signal peptide sequence were synthesized from GeneArt (Life Technologies Carlsbad, CA) and DNA 2.0 (Menlo Park, CA), respectively.

Restriction endonuclease NcoI and XbaI sites were engineered on the 5′ and 3′ termini to facilitate subcloning. Following digestion with NcoI and XbaI the synthetic genes were isolated from an agarose gel and ligated into linearized pPTN200 [19]. The resulting pPTNST108 construct contained the 7S *β*-conglycinin promoter, Tobacco Etch Virus (TEV) translational enhancer, native SEB signal peptide, mutant (L45R, Y89A, Y94A) SEB open reading frame (ORF), and 35S terminator. The construct pPTN764 contained the soybean 11S glycinin promoter and signal peptide sequence, an identical mutant SEB ORF, and 35S terminator elements. Both constructs included a cassette encoding for phosphinothricin acetyltransferase (bar gene) under the control of the nopaline synthase (nos) promoter and terminator elements. Following subcloning, the identity of both constructs was confirmed using multiple restriction digestion analyses. Integrity of the mSEB ORF was verified by double-stranded sequencing (Davis Sequencing, LLC, Davis CA). Soybean transformations were carried out as previously described [20–22].

### 2.2. Preparation of Genomic DNA and PCR

Genomic DNA was prepared from cotyledon tissue using the Maxwell 16 Instrument and the Maxwell Tissue DNA Purification Kit (Promega, Madison, WI). Duplex PCR reactions were carried out using GoTaq Flexi DNA polymerase (Promega, Madison, WI) with the following primers: SEB forward (5′-GGACAAGCGCCTCTTCATCTC-3′), SEB reverse (5′-AGGTACACCTCGATCTTCACG-3′), VSP (vegetative storage protein) forward (5′-GCTTCCACACATGGGAGCAG-3′), and VSP reverse (5′-CCTCTGTGGTCTCCAAGCAG-3′). Following an initial denaturation step at 95°C for 5 minutes the reactions were subjected to 35 cycles comprising denaturation at 95°C for 30 sec, annealing at 52°C for 45 sec, and extension at 72°C for 1 min. PCR products were visualized on 1.5% agarose gels stained with ethidium bromide.

### 2.3. Seed Protein Extracts and Western Blot Analysis

Soluble seed protein was extracted from either seed chips or ground seed powder using an extraction buffer of phosphate-buffered saline (PBS) and sonication for 20 seconds. Samples were clarified from soluble debris by centrifugation and protein concentrations were determined with the Bradford Reagent (Bio-Rad Laboratories, Hercules, CA) using bovine serum albumin (BSA) as a standard.

Soluble protein extracts (3 *μ*g) were subjected to 10% SDS-PAGE under nonreducing conditions. Unless noted, SDS sample buffer did not contain *β*-mercaptoethanol. Samples were transferred in 1x CAPS buffer (N-cyclohexyl-3-aminopropanesulfonic acid, pH 11) containing 10% methanol to Immobilon-P membrane (Millipore, Bedford, MA, USA). Membranes were blocked overnight with 5% nonfat milk in 1XPBS at 4°C, followed by Western analysis with an in-house primary antibody (1 : 5000) and goat anti-rabbit IgG HRP secondary antibody (1 : 5000). Immunodetection was carried out using the SuperSignal West Pico substrate kit (Thermo Scientific, Rockford, IL, USA). For protein visualization, membranes were stained with Coomassie blue for 1 minute followed by destaining.

### 2.4. Quantification of Recombinant Protein in Seed Extracts

Quantification of recombinant soy-mSEB protein expression within seed extracts were determined by Western blot analysis. Protein extracts from a master mix of seed powder consisting of 100 seeds from the T3 generation of ST108 were compared to known amounts of purified recombinant (*E. coli*) mutant SEB standards by Western blot as described above. X-ray films of the results were scanned for densitometric analysis. Integrated density was determined using ImageJ software. A standard curve was plotted using the integrated densities of known mSEB standards. A best-fit standard curve was used to determine the amount of SEB in seed extracts. Theoretical vaccine yields were estimated based on the amount of soluble protein from a starting biomass of 1 L of soy powder (approximately 800 grams) as previously described [23]. Assumptions included 160 mg dry weight of an average soybean seed, 40% seed protein composition, 1.2% expression level for mSEB, and 10 *μ*g for a single human vaccine dose, which is similar to the dose recommended for recombinant hepatitis B surface antigen immunizations [24].

### 2.5. Protein Characterization and N Terminal Sequencing of SEB Fragments

Soybean mSEB was immunoprecipitated using anti-SEB antibodies and protein-A agarose beads (Sigma-Aldrich, St. Louis, MO). The immunoprecipitated protein was electrophoresed on a 10% SDS-PAGE preparative gel in the absence of *β*-mercaptoethanol and soy-mSEB protein was eluted from the gel after Coomassie staining. Eluted protein was dialyzed against PBS and concentrated by using centriplus YM-3 centrifugal filter devices (Millipore, Bedford, MA). Concentrated protein was then mixed with SDS-PAGE sample buffer containing *β*ME, electrophoresed on a 12% SDS-PAGE gel and immobilized onto Immobilon-PVDF membrane. The membranes were stained with Coomassie blue for 1 min, followed by destaining and extensive washes with water. Bands of interest were excised for protein sequencing (Iowa State University protein sequencing facility) of N-terminal amino acids. For signal peptide cleavage prediction, full length amino acid sequences were entered into SignalP 4.1 software [18].

### 2.6. Confocal Microscopy

Whole seed tissue was imbibed for 12 hours in 1XPBS and fixed as described previously [5, 22, 25]. Briefly, sections were permeabilized with 1XPBS containing 0.2% Tween-20 for 10 minutes, followed by blocking in 1XPBS supplemented by 3% BSA overnight at 4°C. Tissue was incubated with rabbit anti-SEB serum (1 : 200) for 4 h at 23°C, followed by incubation with an Alexa Fluor 594 goat anti-rabbit antibody (1 : 200) for 1 h at 23°C. Lastly, tissue sections were incubated with 4′,6-diamidino-2-phenylindole (DAPI) for 5 minutes at 1 : 500 and cover slips were mounted using Gel/Mount aqueous mounting media. Images were collected with a LSM 710 Spectral Confocor 3 Confocal Microscope (Carl Zeiss, Inc.) under 20x magnification and a 405 nm laser to visualize nuclei stained with DAPI in conjunction with a 561 nm laser to collect emitted fluorescence from the Alexa Fluor 594 antibody. Stacks of images (26 optical sections, 20 nm apart) were collected in the Z plane of the specimens and projected to form a single image using the ZEN Light Edition software.

### 2.7. ELISAs

Three different antibodies were used for ELISAs: one used a rabbit polyclonal anti-SEB antibody (generated in house against* E. coli*-derived mSEB) at a concentration of 1 : 500; a second used a commercial HRP-conjugated sheep anti-SEB polyclonal antibody (Abcam number ab15925) at a concentration of 1 : 1000; and a third used a mouse monoclonal anti-SEB (Abcam number ab6064) at a concentration of 1 : 1000. Microtiter plates were coated with 100 ng/well of each protein (soy-mSEB, rSEB, nSEB, or cholera toxin as a control) in 100 *μ*L of 0.1 M bicarbonate buffer (Ph 8.0) at 4°C overnight. Plates were washed in 1XPBS, 0.1% Tween-20, and blocked with 2% BSA for 1 hour. After a second wash detection antibodies were added for 2 hours at room temperature. The in-house anti-SEB ELISA was washed and an anti-rabbit IgG-HRP conjugate was added for 2 hours at room temperature followed by another wash and the addition of the TMB substrate. The commercial HRP conjugated polyclonal anti-SEB ELISA was washed and incubated with TMB substrate (BioFX). The commercial monoclonal anti-SEB ELISA was washed and incubated with HRP-conjugated anti-mouse IgG for 2 hours at room temperature followed by a final wash and the addition of TMB. All reactions were stopped using 0.5 M sulfuric acid and absorbance was read at 405 nm. Absorbance values have not been background subtracted for any of the values given and data are represented as mean ± standard deviation.

### 2.8. Immunization of Mice and Detection of Antibody Titers

Seed extract containing approximately 10 *μ*g of the target soy-mSEB was emulsified in an equal volume of either Complete Freund's adjuvant (primary immunization) or incomplete Freund's adjuvant (booster immunizations). Preimmune serum was collected prior to the first injection and 1 day prior to each booster immunization from groups (*n* = 4) of 4-week-old female BALB/c mice (The Jackson Laboratory, Bar Harbor, ME). Intraperitoneal immunization with seed extract plus adjuvant (10 *μ*g CT) took place on day 0 with boosts on days 14 and 28. To determine anti-SEB titers in sera of immunized mice, microtiter plates were coated with 20 ng/well of native SEB (Toxin Technology, Sarasota Florida) in 100 *μ*L of carbonate buffer at 4°C overnight. Wells were blocked with 3% BSA in PBS. After washing, sera were tested using serial 3-fold dilutions beginning at 1 : 1000 and were incubated for 3 hours at 23°C followed by washes. An HRP-conjugated goat anti-mouse IgG (Southern Biotech, Birmingham, AL) was added for two hours at 23°C. Following washes, plates were incubated with TMB substrate (BioFX) and enzymatic reactions were stopped with the addition of 0.5 M sulfuric acid and absorbance was read at 405 nm. Absorbance values represent serum diluted at 1 : 27,000 and have not been background subtracted and data are represented as mean ± standard deviation.

## 3. Results

### 3.1. Molecular Characterization of Transgenic Events

**Figure 1:**
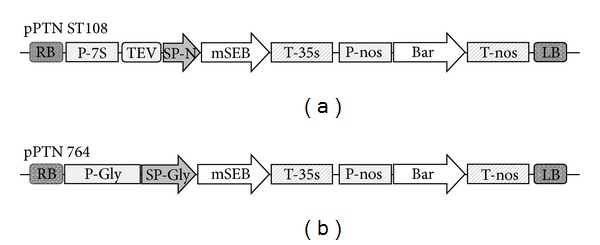
Gene construct design. (a) The pPTN ST108 binary vector used for Agrobacterium-mediated transformation comprising the following regulatory elements: 7S soybean *β*-conglycinin promoter (P-7S), tobacco etch virus translational enhancer element (TEV), native SEB bacterial signal peptide (SP-N), mutant SEB gene (mSEB), and 35S cauliflower mosaic virus terminator element (T-35s) followed by the selectable marker cassette (nopaline synthase promoter (P-nos), phosphinothricin acetyltransferase gene (bar), and nopaline synthase terminator element (T-nos)). (b) The pPTN 764 binary vector contained soybean 11S glycinin promoter (P-Gly), soybean glycinin signal peptide (SP-Gly), mSEB, and T-35S, followed by the selectable marker cassette. Arrows show orientation of cassettes relative to the right border (RB) and left border (LB) sequences.

A synthetic mSEB gene was codon optimized for expression in* Glycine max* and used to create the binary vectors pPTNST108 and pPTN764 (Figure 1). The pPTNST108 construct contains the native* S. aureus* SEB signal peptide sequence and an open reading frame encoding a triple mutant SEB cloned downstream of the soybean *β*-conglycinin promoter. The pPTN764 construct contains an identical mutant SEB open reading frame cloned downstream of the native soybean glycinin promoter and signal peptide elements.

**Figure 2:**
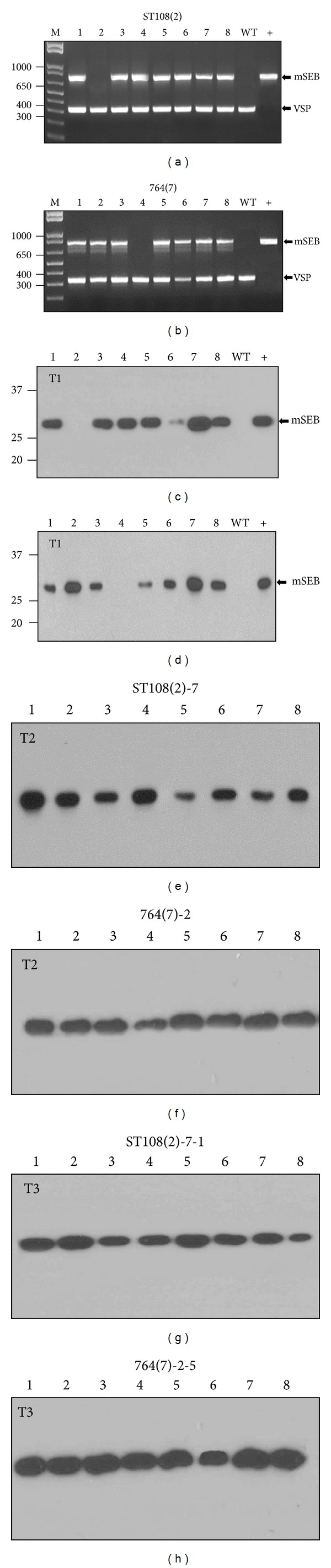
Molecular characterization of soy-mSEB events. (a) and (b) Duplex PCR of 8 T1 progeny from the indicated transformation events. WT: nontransgenic (negative control); +: plasmid DNA (positive control). Arrow shows position of amplified DNA fragments derived from mSEB and vegetative storage protein (VSP). Sizes of molecular weight markers are shown in base pairs. (c) and (d) Western blot of protein derived from the T1 progeny shown in (a) and (b). Arrow indicates soy-mSEB immunoreactive protein. Sizes of molecular weight standards are shown as kDa. (e) and (f) Western blots of T2 progeny from the indicated events. (g) and (h) Western blots of T3 progeny from the indicated events.

*Agrobacterium*-mediated transformation was used to transform soybean somatic embryos. A total of 25 separate transgenic events were obtained using pPTNST108 and 12 transgenic events were obtained using pPTN764. These events were taken to maturity and all appeared to be phenotypically similar to wild-type nontransgenic control plants. A large-scale molecular screen involving duplex PCR and Western analysis was used to identify specific progeny and lines to be moved forward. A representation of the data generated by the molecular screen is shown in Figure 2.

T1 seeds derived from each transformation event were collected and cotyledon chips were prepared from 8 individual seeds. For duplex PCR, genomic DNA was incubated with primers designed to amplify a diagnostic 796 bp soy-mSEB fragment. Primers were also included to duly amplify a 325 bp vegetative storage protein fragment which served as an internal control. For the characterization of ST108 and 764 transformation events shown in Figure 2, duplex PCR identified the mSEB transgene in 7 of the 8 T1 progenies examined (Figures 2(a) and 2(b)). To identify those progenies with detectable mSEB, seed proteins were extracted from each chip, separated under nonreducing SDS-PAGE conditions, and detected by Western analysis. For the representative samples shown in Figure 2, all 7 of the PCR-positive progenies also accumulated immunoreactive protein that was detected by rabbit sera containing anti-SEB polyclonal antibodies (Figures 2(c) and 2(d)). The immunoreactive protein migrated with a MW of ~28 kDa, consistent with the predicted MW of 28.3 kDa for mSEB. The lack of detectable protein in nontransgenic and wild-type seed extracts (negative control) demonstrated the specificity of the antibody for the mSEB epitopes. Recombinant mSEB protein purified from* E. coli* was included on each gel and served as an internal positive control.

Western analyses resulting from a large scale screen of all events revealed that progeny from 18 of the 25 ST108 transgenic events (72%) and 6 of the 12 764 transgenic events (50%) expressed mSEB protein. Based on mSEB expression levels in these experiments, lead progenies were taken to maturity and characterized over multiple generations. The examples shown in Figure 2 represent some of the highest expressing lines that were propagated over several generations and used for subsequent studies. The stability of soy-mSEB was demonstrated by Western analysis in T2 and T3 generations (Figures 2(e), 2(f), 2(g), and 2(h)) and all subsequent generations (data not shown). Southern results preformed on T1 progeny suggested the presence of up to 3 copies of the transgene present at multiple loci.

All plants propagated and taken to maturity were subjected to foliar spray with Ignite 280 SL herbicide to monitor for the expression of the herbicide selectable marker. There was a direct correlation between plants lacking the transgene and severe leaf chlorosis. All plants that contained the transgene and accumulated mSEB showed no visible signs of chlorosis (data not shown).

**Figure 3:**
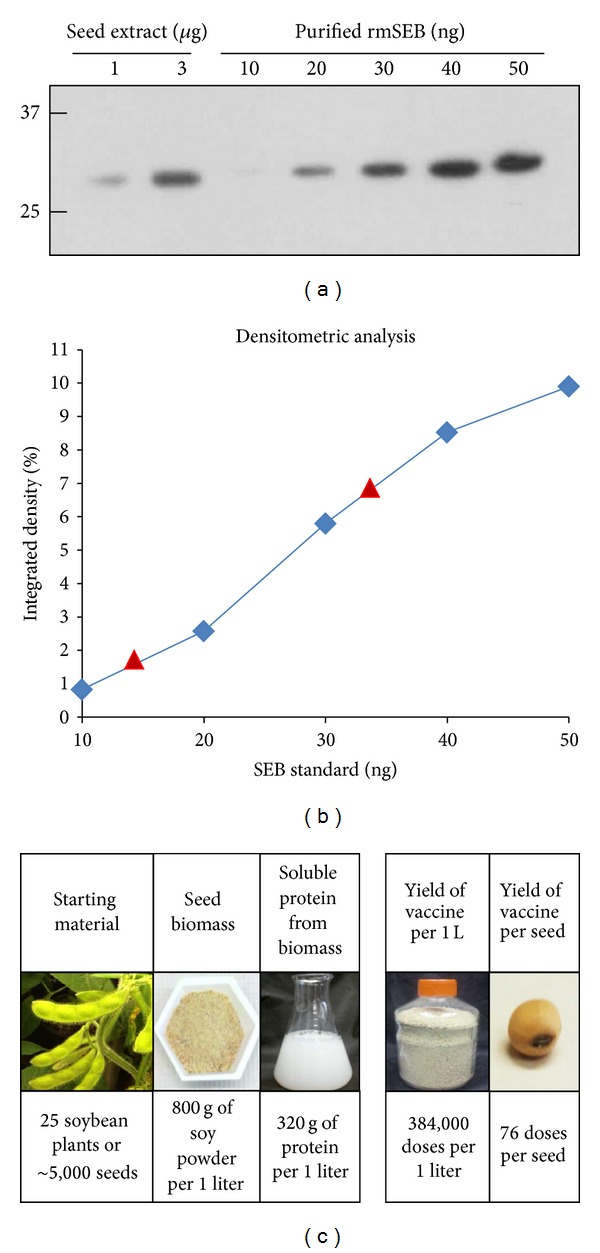
Quantification of soy-mSEB. (a) Known amounts of total seed protein (ST108, T3 generation) and various known amounts of purified* E. coli*-derived mSEB protein (standards) were separated under nonreducing SDS-PAGE and subjected to Western analysis. (b) A standard curve generated from the five known standards following densitometric analysis of the film shown in (a). (c) Chart showing theoretical number of vaccine doses present within a single transgenic soybean seed and in a 1 liter volume of crushed soybean powder. Calculations assume 200 soybeans per plant, 160 mg average seed weight, 40% seed protein content, 1.2% mSEB expression, and a 10 *μ*g human vaccine dose, which is similar to the dose recommended for recombinant hepatitis B surface antigen immunizations [18]. The calculations above do not account for any losses during the purification procedures.

The approximate level of soy-mSEB protein expression was determined by semiquantitative Western analysis. Known amounts of seed protein (extracted from a master powder mix of 100 ST108 homozygous T3 seeds) and purified recombinant mSEB (quantification standards) were used in these experiments (Figure 3(a)). X-ray films of the Western blots were subjected to densitometric examination, and a standard curve was generated. Extrapolation from this curve indicated 13.7 ng mSEB present in 1000 ng total seed protein (1.37% TSP) and 33.6 ng mSEB present in 3000 ng protein (1.12% TSP). Using an average of these numbers, we determined that soy-mSEB represents ~1.2% of total soluble seed protein (Figure 3(b)). These results were also verified by ELISA and imply that an average ST108 soybean seed (160 mg dry weight) with a protein composition of 40% and transgene expression level of 1.2% contains 768 theoretical micrograms of mSEB or 76.8 10 *μ*g human doses of vaccine. This equates to 384,000 vaccine doses produced in seeds produced by ~25 soybean plants (Figure 3(c)).

### 3.2. Soy-mSEB Protein Characterization, N Terminal Sequencing, and Signal Peptide Cleavage

**Figure 4:**
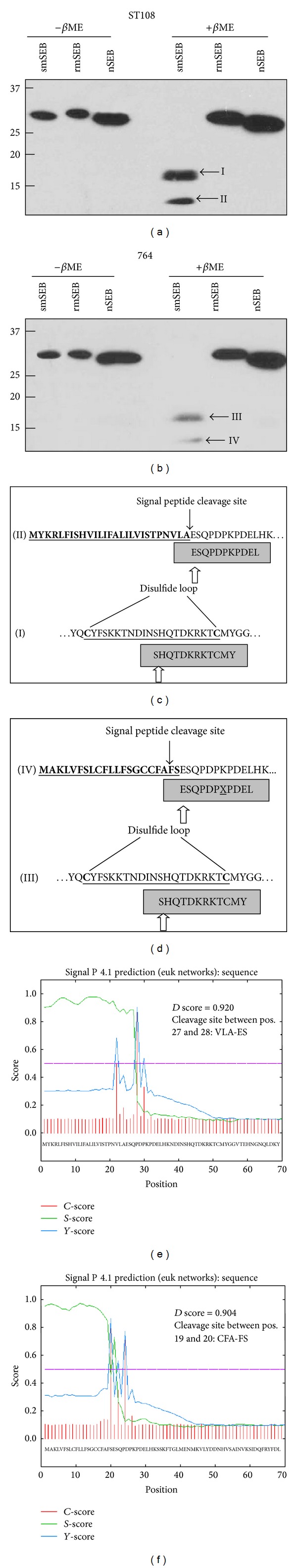
Characterization of soybean-derived mSEB. (a) and (b) Western blot analysis of soy-mSEB,* E. coli*-derived mSEB, and native SEB under nonreducing and reducing conditions. The ST108 soy-mSEB fragments detected under reducing conditions are labeled I and II, while those derived from 764 soy-mSEB are labeled III and IV. (c) and (d) N-terminal sequencing of soy-mSEB fragments detected under reducing conditions. Amino acids identified from N-terminal protein sequencing are shown in shaded boxes and aligned with the relevant portion of the mSEB protein sequence. The bacterial and soybean signal peptide sequences are underlined with bold typeface. Solid arrows indicate the predicted location for signal peptide cleavage and open arrows indicate observed N-termini. (e) and (f) SignalP 4.1 analysis of the ST108 and 764 soy-mSEB amino acid sequences.

Native SEB is a single polypeptide with a known disulfide loop that is essential for mitogenic activity. The cysteines responsible for the disulfide bridge are located at amino acid positions 93 and 113. We noticed that full length soy-mSEB protein could only be detected using nonreducing SDS-PAGE conditions (Figure 2) but not using standard reducing conditions (data not shown). This observation suggested nicking or proteolytic cleavage somewhere within mSEB. To examine this possibility further, soy protein from ST108 and 764 transformation events was compared with* E. coli*-derived mSEB and native SEB protein under reducing and nonreducing conditions. While the inclusion of *β*-mercaptoethanol as a reducing agent did not significantly alter the mobility of the* E. coli*-derived mSEB or native SEB proteins in SDS-PAGE, the inclusion of *β*-mercaptoethanol resulted in the detection of two smaller fragments with mobilities of ~12 and 16 kDa in both soy samples (Figures 4(a) and 4(b)). The appearance and sizes of these fragments are consistent with cleavage within the disulfide loop region. The slight mobility difference observed with the* E. coli*-derived mSEB protein is the result of a C-terminal histidine tag included for purification.

Given the mobility of the two fragments detected under reducing conditions, we predicted that the smaller fragment represented an N-terminal mSEB polypeptide while the larger fragment represented a C-terminal mSEB polypeptide. To map the cleavage sites within soy-mSEB, the larger fragment from both ST108 and 764-derived proteins was subjected to N-terminal protein sequencing. Results from the sequencing experiment identified the N-terminal amino acid residues at the site of cleavage as SHQTDKRKTCMY. This sequence is present within the disulfide loop and confirmed that cleavage of mSEB occurred within this conserved loop region (Figures 4(c) and 4(d)).

Our final characterization of soy-mSEB involved the identification of the N-termini of both mature mSEB proteins. Note that the ST108-derived protein was engineered with a 27-amino-acids bacterial signal peptide while the 764-derived protein was engineered with the 21-amino-acid soybean glycinin signal peptide. The ST108 and 764 mSEB ORF sequences were analyzed using SignalP 4.1 software to predict the presence and location of potential signal peptides. This service predicted cleavage of ST108 between amino acids 27 and 28 and cleavage of 764 between amino acids 19 and 20 (Figures 4(e) and 4(f)). To identify the mature N-terminus of both soy-mSEB proteins, the smaller fragments obtained by treatment with *β*-mercaptoethanol were subjected to N-terminal protein sequencing. In both cases, the N-terminal sequence was identified as ESQPDPKPDEL. This sequence is identical to the N-terminus of mature native SEB. These results verified that the heterologous bacterial signal peptide was accurately recognized and processed by the soybean signal peptidase machinery. This was also the case with the 764 events containing a heterologous glycinin leader peptide sequence.

### 3.3. Soy-mSEB Protein Cellular Localization

**Figure 5:**
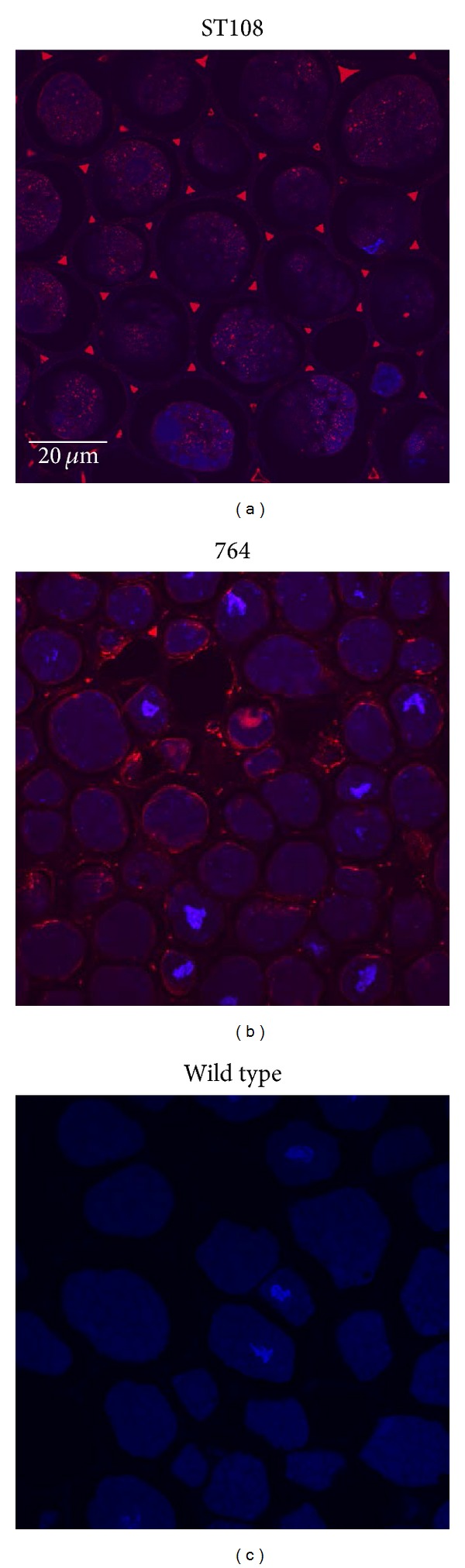
Immunohistochemical detection of soy-mSEB in T2 seeds. (a) ST108 seed section. (b) 764 seed section. (c) Nontransgenic (WT) seed section (control). Red fluorescence represents soy-mSEB protein that is either secreted into apoplastic spaces (ST108) or localized throughout the cell (764). DAPI staining of nuclear material is shown in blue. Samples were viewed at 20x magnification using confocal microscopy, and identical microscope parameters were used for photography of all samples shown.

To determine soy-mSEB localization, immunohistochemistry was carried out on cotyledon tissue using an in-house anti-SEB antibody and an Alexa Fluor 594 goat anti-rabbit IgG secondary antibody. Confocal images show that mSEB derived from ST108 transformation events was secreted into apoplastic spaces (Figure 5(a)) while mSEB derived from 764 transformation events remained intracellular and appeared to be associated with the cell membrane (Figure 5(b)). DAPI staining of nuclear material showed that transgenic protein was also excluded from the nucleoplasm. Fluorescence was not observed in control (nontransgenic) tissues prepared using identical conditions (Figure 5(c)).

### 3.4. Seed Promoter Specificity

**Figure 6:**
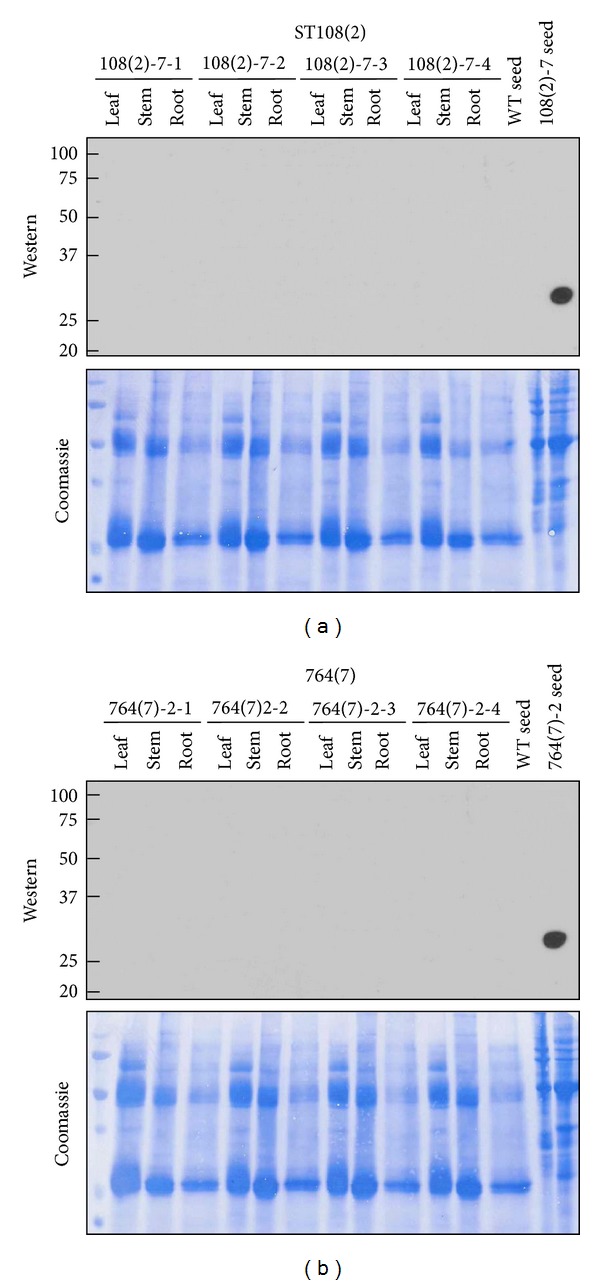
Western blot analysis of promoter specificity. Nonreducing SDS-PAGE conditions were used to separate 10 *μ*g total protein extracted from leaf, stem, and root tissues of the indicated T2 progeny. Equal amounts of T1 seed protein (parent) and nontransgenic (WT) seed protein were also included as controls. Top panels show X-ray film of the resulting Western blots while bottom panels show the blots used in this experiment following staining with Coomassie blue. Sizes of molecular mass standards are shown as kDa. (a) Data for events derived from ST108; (b) data for events derived from 764.

Practical use of soybean as a host for recombinant protein production would involve the harvest of seed and disposal of remaining biomass. To verify that soy-mSEB is present only in seed and not in the leftover biomass, protein was extracted from leaf, stem, and root material and compared with protein derived from master seed powder stocks. Western experiments confirmed that mSEB protein was only detectable in mature seed material and not in leaves, stems, and roots (Figure 6). Coomassie staining of the membranes used in these Western experiments confirmed the presence of plant protein on the blot.

### 3.5. Soy-Derived SEB Is Immunologically Similar to Commercial Forms of SEB

**Figure 7:**
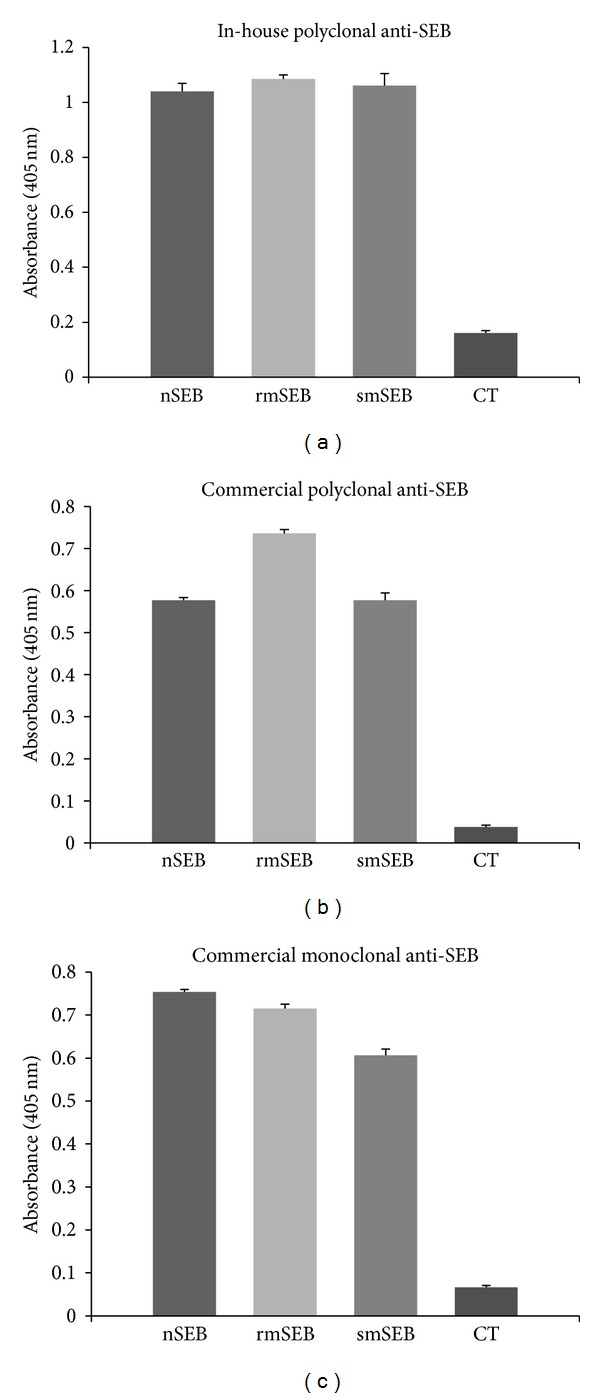
Immunogenicity of SEB proteins. ELISAs were used to determine relative immunogenicities of purified native SEB (nSEB),* E. coli*-derived recombinant mutant SEB (rmSEB), and soy-derived mutant SEB (smSEB) proteins. Cholera toxin (CT) was included as a negative control. 100 ng purified protein was coated in each well. All assays were performed in quadruplicate. (a) ELISA results using an in-house rabbit anti-mSEB polyclonal detection antibody. (b) ELISA results using a commercial sheep anti-SEB polyclonal detection antibody (Abcam number ab15925). (c) ELISA results using a commercial mouse anti-SEB monoclonal detection antibody (Abcam number ab6064). Values shown represent average absorbance values (405 nm). Error bars represent standard deviation.

To evaluate immunogenicity of soy-mSEB relative to* E. coli*-derived mSEB and native SEB, an ELISA was performed using three separate anti-SEB antibodies. Soy-mSEB and* E. coli*-derived mSEB were purified as previously described [23] and purified native SEB was purchased commercially (Toxin Technology, Sarasota Florida). Equal amounts of the three purified proteins, along with cholera toxin (negative control), were coated onto ELISA plates and incubated with different anti-SEB antibodies. Absorbance readings from these ELISAs are shown in Figure 7. An in-house rabbit anti-SEB polyclonal antibody recognized all three proteins similarly (Figure 7(a)). Comparable results were observed in the absorbance readings from ELISAs using a commercially purchased sheep anti-SEB polyclonal antibody (Figure 7(b)). Since polyclonal antibodies are likely to bind both linear and conformational epitopes along the entire length of the SEB protein, these results suggested that soy-mSEB epitopes are intact. A third ELISA was performed using a commercial mouse monoclonal antibody which specifically detects one target epitope on the native SEB protein. Results from this ELISA (Figure 7(c)) were consistent with results from the previous two ELISAs in that absorbance readings for all three SEB proteins were similar. The results obtained here are consistent with the notion that nontoxic soy-mSEB protein is immunologically similar to both* E. coli*-derived mSEB and native SEB.

### 3.6. Immunization of Mice with Soy-mSEB Elicits an Antibody Response

**Figure 8:**
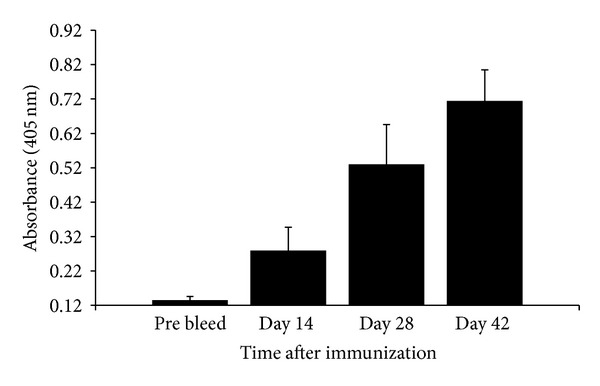
Anti-mSEB titers in mice following immunization. Groups of female BALB/c mice (*n* = 4) were immunized intraperitoneally on day 0 and boosted on days 14 and 28 days with 1 mg transgenic seed extract plus adjuvant. Bleeds were collected just prior to immunization on days 0, 14, and 28, and again on day 42. ELISAs were performed to determine serum IgG anti-mSEB reactivity. Absorbance values (405 nm) represent serum tested at a 1 : 27000 dilution and are presented as mean anti-mSEB titers and error bars represent standard deviation.

To determine whether soy-mSEB could generate specific immunity, groups of mice were administered intraperitoneal injections of transgenic seed protein containing approximately 10 *μ*g of the soy-mSEB vaccine (along with cholera toxin adjuvant) on days 0, 14, and 28. Blood was taken from each animal prior to immunization, and on day 42, and the presence of serum antibodies against soy-mSEB was detected by ELISA (Figure 8). Mice immunized with soy-mSEB showed significant levels of IgG anti-SEB antibody production 14 days after immunization when compared to the prebleed. Antibody titers continued to increase by days 28 and 42 following booster vaccinations. These results demonstrate that the soy-mSEB vaccine candidate was effective in inducing antibodies which recognized native SEB.

## 4. Discussion

Over the past two decades, there has been substantial research on the expression of heterologous proteins in plants as a means to produce biopharmaceuticals. While numerous plant systems have been shown to support expression of heterologous proteins, the soybean has enormous potential with distinct advantages over these other systems. To date soybeans have been engineered to express a variety of therapeutic proteins [5, 26–28]. Soybeans have a high protein content (~40%) making them an excellent host for increased expression and storage of recombinant protein. In the present study we report an expression level of 1.2% of TSP. If one assumes a vaccine dose of 10 *μ*g, as is recommended for recombinant hepatitis B surface antigen immunizations [24], this translates into ~76 theoretical doses of human vaccine in a single seed. Although such calculations represent theoretical protein and do not take into account potential loses during purification they nonetheless represent significantly larger recoverable yield based on biomass when compared with other recombinant protein systems. Another important characteristic of soybeans is that these seeds have evolved as specialized compartments that store proteins for embryo nutrition. Therefore, soybeans possess metabolic adaptations that permit stable and long-term storage of proteins which in turn reduces the requirement for sophisticated and expensive storage conditions. Recombinant proteins expressed in soybean have proven to be stable for years at ambient temperatures [25, 29]. This feature reduces or eliminates the need for a cold chain and allows for recombinant protein production to be a separate event with purification occurring at a later time if needed. Transgenic soybeans can also be used for production of therapeutic formulations that do not require purification. The efficacy of engineered therapeutics in crude soymilk formulations could lead to oral vaccines and other therapies that require little, if any, purification from other seed proteins. These simplified methods for expression, storage, and administration make soybean a cost-effective alternative to existing systems. Successful expression of the mSEB model vaccine antigen in this study demonstrates the practicality of soybean as a viable host for the expression of a vaccine candidate that is biochemically and immunologically functional.

A critical first step for efficient production of a vaccine protein in a recombinant system is to maximize the level of foreign protein expression in an effort to decrease production costs. Soybean seeds are the richest source of protein known, and while constitutive promoters can direct protein expression in seeds [22, 30], it is likely that higher accumulations of target proteins in seeds can be achieved using seed-specific promoters. In this study we used the soybean 7S *β*-conglycinin and 11S glycinin seed storage promoters to target mSEB to seeds. These promoters have also been used by others to successfully express foreign proteins in seed [5, 26, 28, 29]. The use of these promoters allowed us to target soy-mSEB expression to the seed and achieve high levels (1.2% of TSP) of recombinant protein over multiple generations.

In this study we utilized different signal peptide sequences to evaluate subcellular targeting. SEB is a secreted protein which encodes a 27-amino-acid bacterial signal peptide sequence. If this signal peptide is also functional in plants, proteins could potentially be secreted to apoplastic spaces. This location represents a different biochemical environment than intracellular spaces and therefore may impact foreign protein stability. We found that soy-mSEB containing the bacterial signal peptide was accurately processed by the soybean signal peptidase machinery, resulted in a protein with an N-terminus identical to the native protein, and was localized to apoplastic spaces. Since similar levels of soy-mSEB accumulated both extracellularly (with the bacterial signal peptide) and intracellularly (with the soybean glycinin signal peptide) it appears that mSEB does not have a preference for one subcellular location over the other. This is not surprising given that SEB is a highly stable toxin and has evolved its structure to remain stable under a variety of conditions. However, it is possible that apoplastic spaces are the preferred subcellular location for other recombinant proteins, and to this end we have shown that the bacterial SEB signal peptide may be useful in directing such proteins to those spaces. It is interesting to note that the signal peptide from another bacterial secreted protein (*E. coli* labile toxin subunit B, or LT-B) did not appear to have apoplast-targeting capabilities when tested in plants [31]. In that study, expression of chimeric LT-B genes containing either the native LT-B or maize y-zein signal peptide sequences resulted in the unexpected localization of LT-B to starch granules in maize endosperm [31]. In an effort to learn more about the targeting potential of the SEB signal peptide, we are currently testing whether other heterologous proteins can also be localized to apoplastic spaces when the SEB bacterial signal peptide is utilized [31].

Structural studies of SEB have shown the presence of a protruding disulfide loop in this toxin [32]. In the soybean seed environment it appears that this loop is susceptible to nicking by an unknown mechanism. We hypothesize that this nicking occurs either during or shortly after protein synthesis since extraction of seed protein in the presence of protease inhibitors did not prevent cleaved products from being detected (data not shown). Furthermore, incubation of* E. coli*-derived mSEB and native SEB protein preparations with soybean seed extracts did not induce nicking of those proteins, suggesting that seed proteases may not be involved. Previous reports in the literature describe nicking of native SEB [9, 33] and it has been suggested that this nicking is due to enzymatic or chemical hydrolysis during fermentation or purification. Interestingly, this previous work examined native SEB under reducing and nonreducing conditions and detected smaller fragments of SEB when the protein was exposed to reducing conditions; the site of nicking was mapped to the disulfide loop and occurred within 4 amino acids of the site identified [9]. This study also found that some commercial preparations of native SEB were comprised almost entirely of nicked protein while preparations from other vendors showed no evidence of internal cleavage [9]. Although the data presented in Figure 4 showed no signs of nicking in recombinant and native forms of SEB, when these same X-ray films were examined after extended exposure times there were bands present that indicated low levels of nicked SEB in both recombinant and native forms (data not shown). Thus, nicking within the SEB disulfide loop appears to be related to the SEB protein itself and not a phenomenon specific to any expression system. Importantly, the nicked forms of native SEB have been shown to retain full mitogenic activity as long as the disulfide bridge is intact [9]. A phenomenon involving what appears to be proteolytic cleavage of other plant-derived recombinant proteins has also been reported [28] and may be one reason why many recombinant proteins go undetected and associated experiments are deemed unsuccessful.

In order for a soy-based vaccine to be marketable it must be biologically equivalent to (or preferably superior to) an existing vaccine if one is already present in the marketplace. To date there is no commercial vaccine for SEB poisoning; therefore, the mSEB used as a “model” vaccine in this study could also function as an efficacious vaccine if it is shown to be immunogenic and confers protection following challenge with native toxin. To this end we examined the immunmoreactive profile of soy-mSEB and found it to be similar to that of* E. coli*-derived mSEB and native SEB (Figure 7). These observations suggested that immunogenic epitopes throughout soy-mSEB remain intact. The presence of significant levels of anti-SEB antibodies in blood sera of mice occurring within 14 days of immunization alludes to the efficacy of the soy-based mSEB vaccine.

## 5. Conclusions

In this study, a mutated nontoxic version of SEB (soy-mSEB) was produced in transgenic soybean seeds as a highly expressed vaccine. Soy-mSEB was specifically expressed within the soybean seed and was shown to be stably expressed over multiple generations. Soy-mSEB was successfully localized both intra- and extracellularly and accumulated equally in both subcellular locations. Soy-derived mSEB was shown to be biochemically and immunologically similar to recombinant and wild-type commercial forms of SEB. Additionally, functionality of the soy-mSEB as a vaccine antigen was demonstrated using mice which produced anti-SEB titers in blood serum after vaccination with soy-mSEB. Taken together, these results show the efficacy of soy-derived mSEB and demonstrate the potential for soybean as a platform technology to produce pharmaceutical proteins.

To further explore the effectiveness of the soy-mSEB vaccine, current studies are underway to determine whether immunization with purified soy-mSEB confers protection in an animal model when challenged with the native toxin, and if so, whether such protection is comparable to or superior to protection obtained by vaccination with other recombinant forms of mSEB.
